# Crystal Structure of Calcium Binding Protein-5 from *Entamoeba histolytica* and Its Involvement in Initiation of Phagocytosis of Human Erythrocytes

**DOI:** 10.1371/journal.ppat.1004532

**Published:** 2014-12-11

**Authors:** Sanjeev Kumar, Saima Aslam, Mohit Mazumder, Pradeep Dahiya, Aruna Murmu, Babu A. Manjasetty, Rana Zaidi, Alok Bhattacharya, S. Gourinath

**Affiliations:** 1 School of Life Sciences, Jawaharlal Nehru University, New Delhi, India; 2 Department of Biochemistry, Jamia Hamdard, New Delhi, India; 3 Plant Mediator Lab, National Institute of Plant & Genome Research, New Delhi, Delhi, India; 4 European Molecular Biology Laboratory, Grenoble Outstation, France; 5 Unit for Virus Host-Cell Interactions, Université Grenoble Alpes - EMBL-CNRS, France; University of Virginia Health System, United States of America

## Abstract

*Entamoeba histolytica* is the etiological agent of human amoebic colitis and liver abscess, and causes a high level of morbidity and mortality worldwide, particularly in developing countries. There are a number of studies that have shown a crucial role for Ca^2+^ and its binding protein in amoebic biology. EhCaBP5 is one of the EF hand calcium-binding proteins of *E. histolytica.* We have determined the crystal structure of EhCaBP5 at 1.9 Å resolution in the Ca^2+^-bound state, which shows an unconventional mode of Ca^2+^ binding involving coordination to a closed yet canonical EF-hand motif. Structurally, EhCaBP5 is more similar to the essential light chain of myosin than to Calmodulin despite its somewhat greater sequence identity with Calmodulin. This structure-based analysis suggests that EhCaBP5 could be a light chain of myosin. Surface plasmon resonance studies confirmed this hypothesis, and in particular showed that EhCaBP5 interacts with the IQ motif of myosin 1B in calcium independent manner. It also appears from modelling of the EhCaBP5-IQ motif complex that EhCaBP5 undergoes a structural change in order to bind the IQ motif of myosin. This specific interaction was further confirmed by the observation that EhCaBP5 and myosin 1B are colocalized in *E. histolytica* during phagocytic cup formation. Immunoprecipitation of EhCaBP5 from total *E. histolytica* cellular extract also pulls out myosin 1B and this interaction was confirmed to be Ca^2+^ independent. Confocal imaging of *E. histolytica* showed that EhCaBP5 and myosin 1B are part of phagosomes. Overexpression of EhCaBP5 increases slight rate (∼20%) of phagosome formation, while suppression reduces the rate drastically (∼55%). Taken together, these experiments indicate that EhCaBP5 is likely to be the light chain of myosin 1B. Interestingly, EhCaBP5 is not present in the phagosome after its formation suggesting EhCaBP5 may be playing a regulatory role.

## Author Summary

*Entamoeba histolytica* is the etiologic agent of amoebiasis, a major cause of morbidity and mortality in developing countries. The genome of this organism encodes 27 EF-hand containing calcium binding proteins suggesting an intricate Ca^2+^ signalling system that plays crucial role in phagocytosis and pathogenesis. Calcium binding protein-5 (EhCaBP5) is one of these CaBPs that displays sequence similarity with Calmodulin (CaM) but has only two possible calcium binding EF-hand loops in two separate domains. Interestingly crystal structure of EhCaPB5 showed more structural similarity with essential light chain (ELC) of myosin than that of CaM. The binding studies of EhCaBP5 with IQ motif peptides of myosins, showed that it interacts with IQ motif of unconventional Myosin IB. A number of experiments were carried out to show that EhCaBP5 indeed binds myosin IB and that this binding is Ca^2+^ independent. We also show here that EhCaBP5 participates in erythrophagocytosis and that its role in phagocytosis is different from that of EhCaBP3, another myosin 1B interacting calcium binding protein of E. histolytica. Our results presented here and in a number of other reports point towards a unique phagocytic pathway involving a number of calcium binding proteins in *E. histolytica.*

## Introduction

*Entamoeba histolytica* is the etiological agent of amoebiasis (intestinal as well as extra-intestinal), which results in a high level of morbidity and mortality worldwide, particularly in developing countries [1], [2]. A number of studies have shown that Ca^2+^ and its binding proteins are centrally involved in amoebic pathogenesis and that cytolytic activity can be blocked by Ca^2+^ channel blockers or treatment with EGTA [3]. Genomic analysis of *E. histolytica* indicates the presence of 27 genes encoding multiple EF-hand calcium-binding proteins (CaBPs) [4]. The presence of such a large number of CaBPs suggests that this organism has a complex and extensive calcium signalling system [4].

One of the Ca^2+^ sensing proteins of *E. histolytica*, EhCaBP1, has been extensively characterised, both structurally and functionally. EhCaBP1 was found to be involved in cytoskeleton dynamics and is associated with phagocytic cup formation in a Ca^2+^ independent manner [5], [6]. The binding of Ca^2+^ to EhCaBP1 is necessary for the transition of phagocytic cups to phagosomes [7]. EhCaBP1 is recruited to phagocytic cups by the novel protein kinase EhC2PK [8]. The crystal structure of EhCaBP1 shows an unusual trimeric arrangement of EF-hand motifs [9]. The structure of the N-terminal lobe of EhCaBP1 displays a similar trimeric organization of EF-hand motifs as observed in the full length molecule. Lowering the pH to below physiological levels was shown to cause a trimer to monomer transition [10]. Moreover, various metal ions have been shown to impart flexibility and plasticity to the EF-hand motifs of EhCaBP1 [11].

We (and others) are systematically investigating the structure-function relationship of other calcium binding proteins of *E. histolytica* as well in order to understand their roles in amoebic biology and pathogenesis. Recently, an NMR structure of the calmodulin-like calcium-binding protein EhCaBP3 has been reported [12]. The N-terminal half of the molecule displays a structure similar to that of CaM, but no structure was derived for the C-terminal half of the molecule [12]. EhCaBP3 was found to be involved in the regulation of phagocytosis and cytoskeleton dynamics [13]. In addition to the studies of EhCaBP1 and EhCaBP3, we have collected (reported) preliminary crystallographic data of EhCaBP2 [14].

Sequence analysis of the calcium binding protein 5 from *E. histolytica* (EhCaBP5) indicates that its size (16.3 kDa) and secondary structural arrangement are similar to those of CaM like proteins but it also suggests the presence of two calcium binding loops in two separate lobes. In CaM like proteins, two functional calcium binding EF-hand motifs usually exist side by side, and participate in calcium dependent target binding. The possible existence of two calcium binding sites in two separate lobes in EhCaBP5 prompted us to study the structure and function of this protein.

We previously crystallized EhCaBP5 [15], and here we report the structure determination and results from functional studies of this protein. We have determined the crystal structure in the calcium bound state at 1.9 Å resolution and have shown that EhCaBP5 interacts with the unconventional myosin (myosin 1B), by surface plasmon resonance (SPR). This interaction was further confirmed by a pull down assay and cellular co-localization using confocal microscopy. The results suggest that EhCaBP5 is involved in phagosome formation through interaction with myosin 1B. We conclude that phagocytosis in *E. histolytica* is regulated by a number of CaBPs including EhCaBP5 that regulates cytoskeletal dynamics and phagocytic cup formation with the help of myosin 1B, a process not observed in any other organism.

## Results

### Structure of EhCaBP5

Molecular replacement with several calmodulin like proteins failed to give any solution, even though EhCaBP5 is, for example, 29% identical to potato calmodulin [16]. Instead, Se-Met labelled EhCaBP5 crystals were used to provide experimental phases and determine the structure (see Methods for details). There is only one molecule present in the asymmetric unit. The final refined structure contains 131 residues, one calcium ion, 40 waters and 3 acetates; six residues from the N-terminus could not be modelled due to missing electron density.

**Figure 1:**
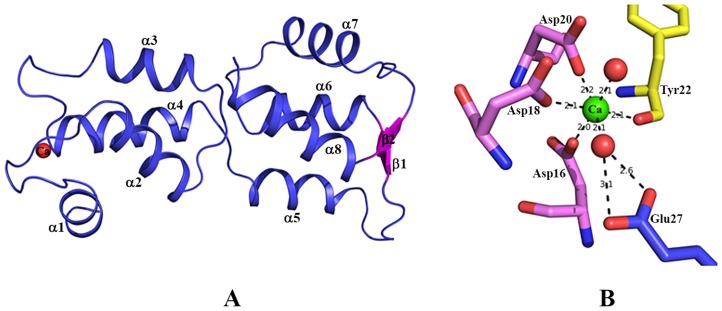
Structure of EhCaBP5. (A) Overall structure of *Entamoeba histolytica* calcium binding protein-5 (EhCaBP5) at 1.9 Å resolution, shown as a ribbon diagram. All of the alpha helices (α1 to α8) are in blue, the small antiparallel β-sheet (β1 and β2) is represented in pink, and the bound Ca^2+^ is shown as a red sphere. (B) Ca^2+^ coordination of EhCaBP5: The unconventional mode of Ca^2+^binding by the EF hand motif of EhCaBP5 (see text for details).

Overall, the molecule is divided in two globular lobes, where each lobe has four alpha helices connected by loops. The N-terminal lobe has one EF-hand motif with a calcium ion bound to the loop between two helices. The C-terminal lobe has two small anti-parallel beta strands along with four loops (Fig. 1A). Signature residues of an additional EF-hand motif are found in the C-terminal lobe (residues87 to 98). In the crystal structure these residues form a loop between two helices similar to that of a typical EF-hand but density for Ca^2+^ is not observed. Consistent with the crystals structure, only one site in EhCaBP5 was found to bind Ca^2+^ by ITC [17]. Taken together, these results suggest that the C-terminal lobe of EhCaBP5 cannot bind Ca^2+^. Nevertheless, the overall conformation of the Ca^2+^ bound N-terminal lobe is similar to that of the Ca^2+^ free C-terminal lobe, with an r.m.s.d.of 1.9 Å between these lobes.

### Calcium coordination in the EF-hand motif of EhCaBP5

The coordination of Ca^2+^ observed in the EhCaBP5 crystal structure has only been seen in essential light chains (ELC) of myosin [18], [19] and a calmodulin mutant [20]. In EhCaBP5, the Ca^2+^ is coordinated by one carboxyl oxygen each of residues Asp_16_, Asp_18_, and Asp_20_, the hydroxyl oxygen of Tyr_22_, and two water molecules (instead of one, as observed in CaM). The extra water also binds to the 12^th^ position glutamate of the EF hand loop; this residue is usually critical in directly coordinating Ca^2+^, but, in EhCaBP5, it is too far away, at a distance of about 4.1 Å, and cannot directly coordinate instead it coordinates the Ca^2+^ via the intervening water molecule (Fig. 1B). Overall, the Ca^2+^coordination geometry is octahedral in EhCaBP5, instead of being pentagonal bipyramidal as is typically found in CaM. The octahedral geometry is more similar to that of Mg^2+^ coordinating in other calmodulin like proteins and essential light chain proteins.

### Comparison with calmodulin and myosin essential light chains (ELC)

Among calcium binding proteins, EhCaBP5 displays the highest sequence similarity with potato CaM, with approximately 29% sequence identity. Moreover, EhCaBP5 is composed of two globular lobes similar to that of CaM. EhCaBP5, however, differs from CaM regarding lobe composition. The central linker of EhCaBP5 is not a straight helix, but is broken in the middle, resulting in four separate helices in each lobe (Fig. 1A). Both lobes interact with each other and appear as they are one over another. These differences may help explain the failure of the molecular replacement method for solving the EhCaBP5 structure as described above.

**Figure 2:**
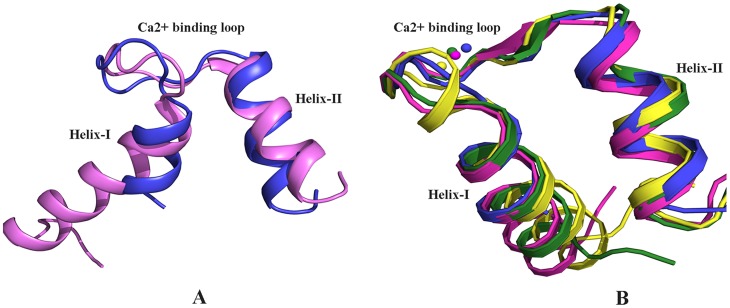
Structural comparison. A) Alignment of the Ca^2+^ bound EF-hand motif of EhCaBP5 (blue) with that of Potato CaM (pink, pdb code 1RFJ). The r.m.s.d. of alignment is 2.38 Å. The interhelical angles of the EF-hand motifs of EhCaBP5 and CaM are 64.3 Å and 89.3 Å, respectively, indicative in turn of the closed and open conformations. (B) Alignment of the Ca^2+^ bound EF-hand motif of EhCaBP5 (blue) with that of squid ELC (yellow, pdb code 1QVI), physarum ELC (green, pdb code 2BLO), and a mutant CaM (pink, pdb code 1Y6W). The rmsd's of the alignments are1.6 Å, 1.3 Å and 1.08 Å, respectively.

There is also a striking difference between the (Ca^2+^ bound) EF-hand motifs of EhCaBP5 and that of CaM. The CaM EF-hand motif (for example that in PDB code 1RFJ) [16] adopts an open conformation after binding of Ca^2+^, whereas the Ca^2+^ bound EF hand motif of EhCaBP5 is in a closed conformation. The r.m.s.d. between these EF-hand motifs is indeed relatively large, at 2.38 Å, and the interhelical angles of the EF-hand motifs of EhCaBP5 and CaM are 64.3 degrees and 89.3 degrees respectively, indicative of the closed and open conformations respectively (Fig. 2A).

The overall structure of EhCaBP5 is more similar to that of the essential light chain (ELC) of myosin than to CaM. Both EhCaBP5 and the ELCs have two 4-helix globular lobes connected by a small loop. Comparison with other Ca^2+^ binding proteins and ELC structures shows that the Ca^2+^ bound EF-hand motif of EhCaBP5 is particularly similar to that of squid myosin ELC [18] (rmsd  = 1.6 Å) and to that of physarum myosin ELC [19] (rmsd  = 1.3 Å), adopting a closed conformation in each structure (Fig. 2B). In these two ELC structures, as well as in EhCaBP5, only one bound Ca^2+^ is seen in the N-terminal lobe. The closed EF-hand motif of squid ELC is due to the presence of an extra turn in the first helix and additional stabilizing interactions with the RLC [18]. In contrast, the EF-hand of the Physarum ELC structure lacks this extra helical turn, and in fact has a fully canonical loop structure, but is still observed in a closed state [19]. Such an unconventional mode of Ca^2+^ binding (combination of closed EF-hand motif and canonical residues) also occurs in EhCaBP5. Also note that the conformation of the Ca^2+^ bound EF-hand motif of EhCaBP5 is very similar to that of the first EF hand motif of the trapped intermediate state of a CaM mutant (rmsd  = 1.08 Å; Fig. 2B); in this mutant, the EF-hand motif is locked in a closed conformation by a disulphide bond, even though Ca^2+^ is bound [20].

Sequence alignment suggests that the residues of the first and second helices of the EhCaBP5 EF-hand motif are more hydrophobic than those in CaM and Physarum ELC. The extensive hydrophobic interaction between these two helices could be the reason for the observed closed state of the lobe and water mediated Ca^2+^coordination or intermediate state in EhCaBP5. This indicates that Ca^2+^ binding energy is not sufficient to open the hydrophobic pocket.

### EhCaBP5 interacts with myosin 1B

**Figure 3:**
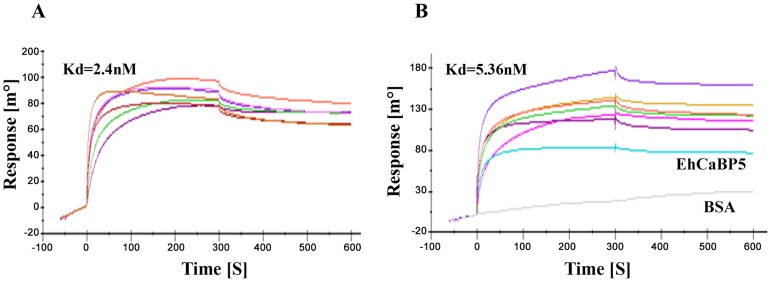
Binding of EhCaBP5 with IQ motif. SPR Sensograms representing interaction between myosin 1B IQ motif peptide (IQKAWKGYRNRKR) and EhCaBP5 in presence of Ca^2+^ (3A) and absence of Ca^2+^ (presence of 5 mM EGTA) (3B). Different concentrations of CaBP5 were injected onto the sensor chip to calculate the dissociation constant (Kd). BSA was also injected at concentration 750 nM (Fig. 3B, grey colour).

We tested whether EhCaBP5 can bind peptides representing IQ motif derived from myosin sequences as EhCaBP5 resembles ELC's, and since one of the two IQ motifs of myosin II binds ELC. *E. histolytica* genome encodes two myosins, myosin 1B and myosin II, containing one and two IQ motifs respectively. SPR was employed to carry out binding assays using the IQ motif peptide from myosin 1B and from ELC binding IQ motif of myosin II. The results indicate that EhCaBP5 does not bind the myosin II IQ motif while it does interact with the IQ motif from myosin 1B (Kd = 2.4 nM) (Fig. 3A). To check the role of Ca^2+^ in this interaction, we performed this experiment in absence of Ca^2+^. The result showed binding with Kd of 5.4 nM, suggesting that it takes place even in the absence of Ca^2+^ but with reduced affinity (Fig. 3B). The binding of EhCaBP5 to myosin 1B IQ motif peptide is very specific, as neither BSA (Fig. 3B) nor EhCaBP3 (Figure S1) was found to interact with immobilized IQ motif peptide. It is possible that EhCaBP3 interacts with myosin 1B through non IQ motif region. This binding seems to be stronger than CaM-Myo1c IQ motif interaction [21].

**Figure 4:**
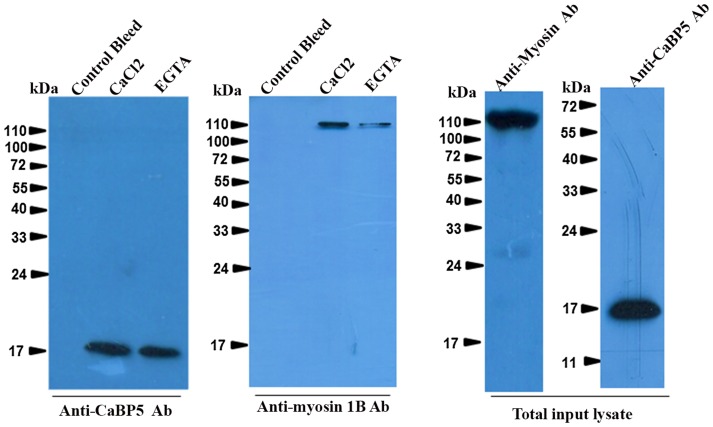
Interaction of EhCaBP5 with myosin 1B. Total (800 µg) *E. histolytica* lysate was incubated with Sepharose-anti-CaBP5 antibody conjugate for 6 h at 4°C with shaking. The beads were then washed and the bound material was then eluted and analysed by western blotting followed by immunostained with anti-myosin 1B antibody raised in rabbit. The blot was reprobed with anti-CaBP5 antibody raised in mice. The total input lysate was also probed for the presence of EhCaBP5 and myosin 1B by their respective antibodies.

In order to further confirm the interaction between EhCaBP5 and myosin 1B, we carried out co-immuno precipitation using immobilized anti-EhCaBP5 antibody from the total cell lysate. The antibody precipitated myosin 1B along with EhCaBP5 even in the presence of EGTA confirming that Ca^2+^ is not required for EhCaBP5 to bind myosin 1B (Fig. 4).

### Localization of EhCaBP5 in *E. histolytica*

**Figure 5:**
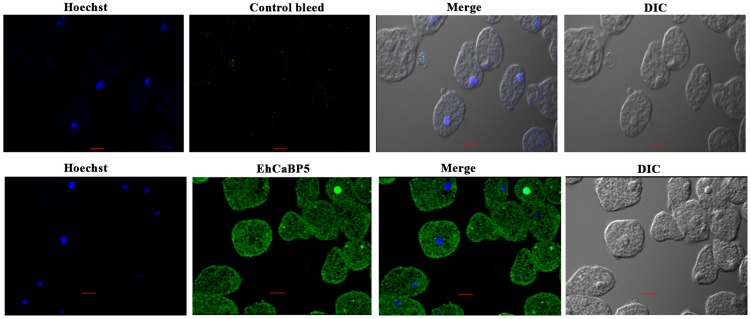
Immunolocalization of EhCaBP5 in *E. histolytica*. Trophozoites grown for 48 h were transferred to pre-warmed coverslips for 10 min at 37°C. The cells were then fixed with 3.7% paraformaldehde/PBS, permeabilized with 0.1% triton X-100/PBS and then stained with anti-EhCaBP5 antibody followed by secondary antibody Alexa 488. Hoechst was used to stain the nuclei. Bar represents 10 µm. Magnification 60X.

**Figure 6:**
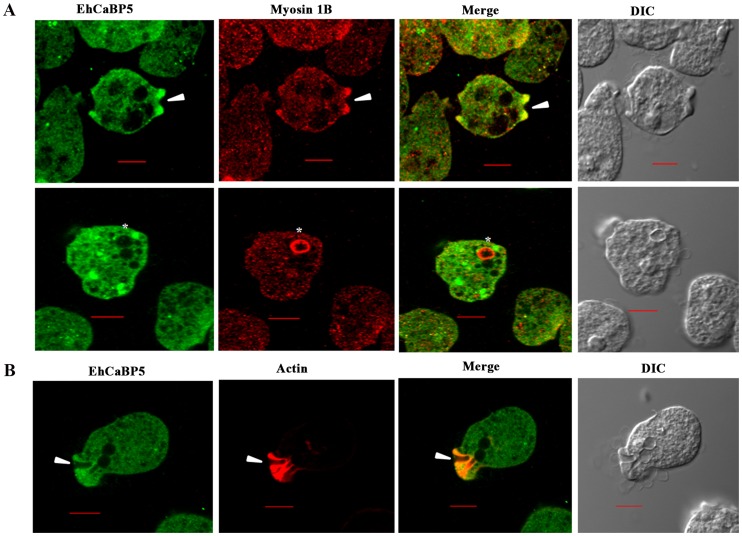
Distribution of EhCaBP5, myosin 1B and actin in *E. histolytica* during erythrophagocytosis. (A) Co- localization of EhCaBP5 and myosin 1B in *E. histolytica* cells during erythrophagocytosis. Cells were grown for 48 h and incubated with RBCs for 10 min at 37°C. The cells were then fixed and immunostained with anti- EhCaBP5 and anti-myosin 1B antibodies followed by Alexa-488 (green) and Alexa-555 (red) secondary antibodies. Arrow head depicts the co- localization of CaBP5 and myosin 1B in the phagocytic cup (upper panel) and an asterisk mark shows the absence of EhCaBP5 in the phagosome. (B) Co-localization of EhCaBP5 with F-actin. Trophozoites were stained with anti-CaBP5 antibody and TRITC-phalloidin (red) was used to stain the F-actin. The secondary antibody used for EhCaBP5 was Alexa-488 (green). Bar represents 10 µm. (DIC, differential interference contrast).

Immunofluorescence was used to investigate the localization of EhCaBP5 in proliferating amoebic cells. The results are shown in Fig. 5. EhCaBP5 was found in the cytoplasm and no fluorescence signal was observed in the nucleus unlike EhCaBP3. Since myosin 1B was shown to be involved in erythrophagocytosis [22], further experiments were carried out to investigate whether EhCaBP5 is also involved in phagocytosis. We monitored EhCaBP5 localization during RBC uptake by *E. histolytica* to check the involvement of EhCaBP5 in phagocytosis. EhCaBP5 and myosin 1B were found in phagocytic cups based on analysis of fluorescence signals (Fig. 6A, upper panel). Interestingly while myosin 1B was also found in the phagosomes (denoted by asterisk) as expected, EhCaBP5 was not seen, suggesting that EhCaBP5 is involved in the initiation phase of phagocytosis (Fig. 6A, lower panel). Enrichment of actin was also observed in the phagocytic cups, as expected, and the superimposition of both EhCaBP5 and actin signals suggested that both proteins are co-localized in the phagocytic cups (Fig. 6B). The results clearly show that EhCaBP5, myosin 1B and actin are all colocalized in phagocytic cups. To check whether actin and EhCaBP5 also interact *in vitro*, we performed a co-sedimentation assay using F-actin and EhCaBP5. No significant amount of EhCaBP5 was observed in the pellet fraction containing F-actin unlike EhCaBP3, suggesting that participation of EhCaBP5 in phagocytosis follows a different path than that of EhCaBP3. The results together clearly showed that EhCaBP5 is involved in amoebic phagocytosis by directly interacting with IQ motif of myosin 1B.

### Down regulation of EhCaBP5

**Figure 7:**
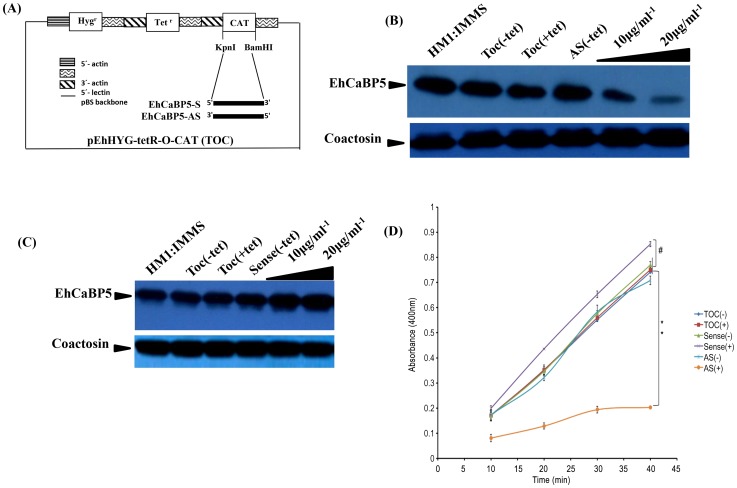
Down regulation of EhCaBP5 reduces the rate of phagocytosis. (A) Schematic representation of sense and anti-sense construct. EhCaBP5 was cloned in sense and anti-sense orientation in BamH1 and Kpn1 site of pEhHyg-TetR-O-CAT vector. (B, C) Western blot analysis of anti-sense (B) and Sense EhCaBP5 (C). Thirty microgram of lysate from sense and forty microgram of anti-sense cells in presence and absence of tetracycline were loaded to each lane. HM1 was used as a positive control and coactosin as a loading control. (D) RBC uptake assay performed with cells expressing sense and anti-sense constructs in the presence and absence of tetracycline. The experiment was repeated three times independently in triplicates. Comparisons were made with respect to cells vector alone or cells without addition of tetracycline. Statistical significance was determined by paired- t test. P-values for ** is P<0.001, #P>0.05.

**Figure 8:**
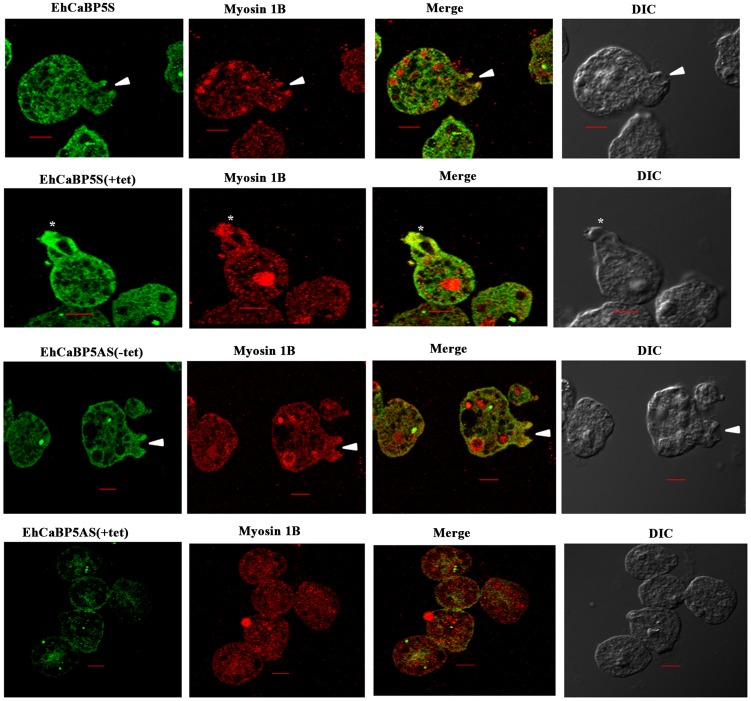
Erythrophagocytosis of cells overexpressing EhCaBP5 antisense and sense RNA constructs in the presence and the absence of tetracycline. Amoebic cells overexpressing EhCaBP5S and EhCaBP5AS with and without tetracycline were incubated with RBC for 10 min. The cells were then stained with Alexa 488(EhCaBP5) and myosin 1B (red). Solid arrow depicts the phagocytic cup and asterisk shows the phagosome. (scale bar, 10 µm; DIC, differential interference contrast).

The results shown so far suggest strongly that EhCaBP5 is involved in amoebic phagocytosis. In order to show if it is required, we determined erythrophagocytosis levels in cells where EhCaBP5 expression was down regulated by specific antisense RNA [23]. The vector used and details of different constructs are shown in Fig. 7A. The level of EhCaBP5 was significantly (62%) reduced on tetracycline addition in the cells carrying antisense construct (EhCaBP5AS) as compared to the cells carrying only the vector (Fig. 7B). This effect was specific as a control coactosin levels were monitored and the amount of coactosin did not change. EhCoactin is F-actin stabilizing protein, recently we have shown that overexpression of this protein results in arrest of phygocytic cup formation [24]. EhCaBP5 gene was over expressed using the cloned gene in the sense orientation (EhCaBP5S) the amount of EhCaBP5 increased by 25% in the presence of 10 µg/ml of tetracycline (Fig. 7C). *E.histolytica* cells carrying all these constructs were then checked for erythrophagocytosis using a spectrophotometric assay. Cells expressing EhCaBP5 antisense RNA (that is, in the presence of tetracycline) displayed reduced (55%) rate of phagocytosis as compared with cells carrying only the vector in the presence of tetracycline and the cells carrying EhCaBP5 antisense construct in the absence of tetracycline. On over expression of EhCaBP5 with the help of a sense construct, an increase of nearly 20% in erythrophagocytosis was observed as compared to cells without tetracycline or vector containing cells in the presence of tetracycline (Fig. 7D). Phagocytic cup formation also showed similar pattern as that of phagocytic rates. The results are shown in Fig. 8. Cup formation was reduced and delayed in cells expressing anti-sense RNA. A few cups were seen only after 10 min of incubation with RBC in antisense cells. In control cells generally cups were visible within a minute after addition of RBC.

**Figure 9:**
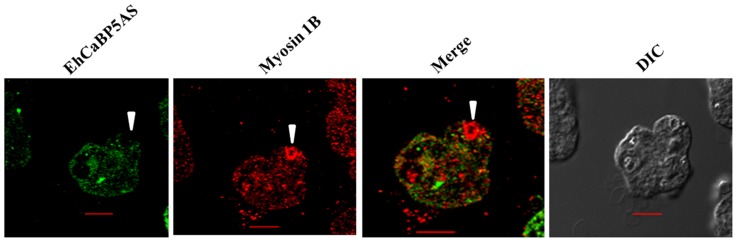
Erythrophagocytosis of amoebic cells overexpressing EhCaBP5 antisense RNA in presence of tetracycline. Amoebic cells were incubated with RBC for 15 min. The cells were then stained with Alexa 488 (EhCaBP5) and myosin 1B (red). Solid arrow heads represent the closure of phagocytic cup. (scale bar, 10 µm; DIC, differential interference contrast).

We also tested if EhCaBP5 is needed in the recruitment of myosin 1B. This was done by imaging myosin 1B in actively phagocytosing cells that are expressing anti-sense RNA of EhCaBP5. There was no significant effect on myosin 1B staining in cells with reduced concentration of EhCaBP5 that is in presence of tetracycline in antisense construct carrying cells suggesting that EhCaBP5 is not needed in myosin 1B recruitment (Fig. 9).

### Model of the EhCaBP5-IQ motif complex

**Figure 10:**
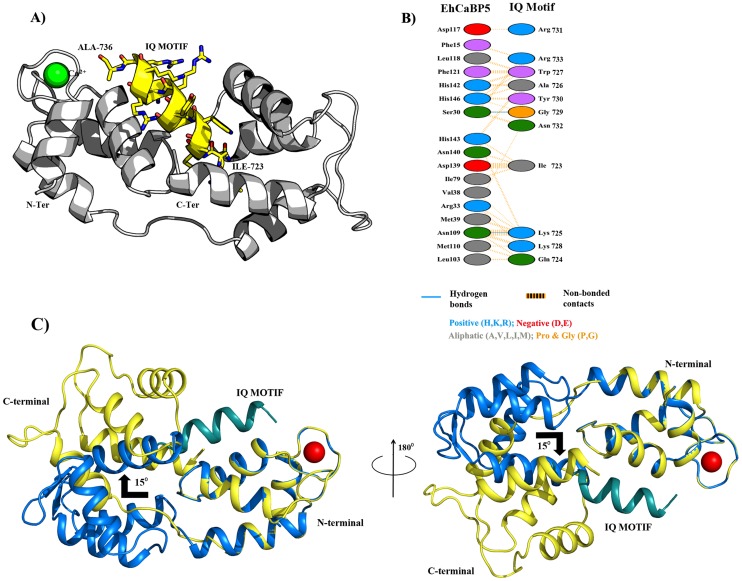
Protein-peptide Interaction. A) Rosetta-docked lowest energy model of EhCaBP5 (grey ribbon) bound with an IQ motif (yellow ribbon for backbone and sticks for side chains). B) Schematic representation of the contacts between EhCaBP5 and the peptide. The interaction shown by dotted orange lines are the non-bonded interactions, in which the width of the striped line is proportional to the number of atomic contacts. A blue line between any two residues indicates hydrogen bond interactions. C) Superimposition of the native EhCaBP5 crystal structure on the model of peptide-bound EhCaBP5 shows a predicted 15° rotation of the C-lobe relative the N-lobe upon binding of the peptide.

A theoretical model of the EhCaBP5-Myosin 1B IQ motif complex was generated using molecular docking simulations in order to predict conformational consequences of peptide binding as well as the details about the interaction between the protein and myosin 1B IQ motif peptide. The EhCaBP5 C-terminal domain adopts a more open conformation in the peptide bound model compared to native EhCaBP5 in absence of the peptide. It appears from our model that EhCaBP5 accommodates the IQ-motif peptide in the cleft, and N- and C-terminal lobes of EhCaBP5 move apart to wrap around the peptide. The model suggest that N-terminus of the peptide may interacts with the C-terminal lobe of EhCaBP5, and the C-terminus of the peptide binds to the N-terminal domain of EhCaBP5 (Fig. 10A). Probable interface residues of EhCaBP5 that may involve in interaction are F-15, G-17, E-27, S-30, R-33, M-39 and D-117 with that of R-731, G-729, N-732, K-725, K-728 and R733 of the myosin peptide (Fig. 10B).

### Comparison of Apo and complex structure of EhCaBP5

Superimposition of the native EhCaBP5 structure on the EhCaBP5-IQ motif model (Fig. 10C) indicates no global change upon peptide binding. The conformations of the N-terminal domains of the crystal structure and the Rosetta docked model are nearly identical, with an r.m.s.d. of 0.072 Å. The simulations, however, yielded about 15 degree reorientation of the C-terminal domain related to N-terminal domain, compared to the native structure, to accommodate the peptide resulting in a stretching out of the central loop connecting the two domains (Fig. 10C). This has led to a change in overall length from 47.7 Å to 55.1 Å between native and peptide bound structures respectively. Moreover, the r.m.s.d. between the C-terminal domains of the native crystal structure and peptide bound model is 0.601 Å, reflecting greater predicted conformational changes within the C-terminal domain, as compared to within the N-terminal domain, upon binding of the peptide. These changes result in reorientations of the helices that help the molecule to have an open conformation needed to bind the peptide.

### Model validation

**Figure 11:**
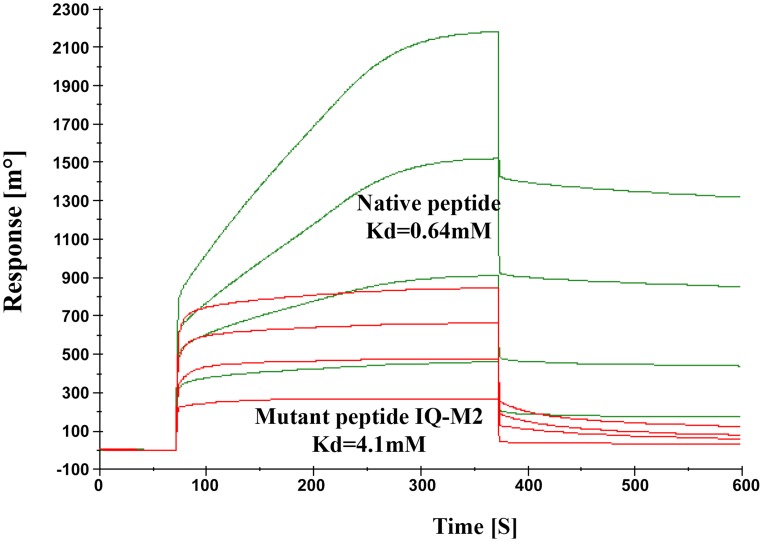
Model Validation. SPR Sensogram represents the binding of Native IQ motif peptide (green) and Mutant IQ-M2 peptide (red) to EhCaBP5 immobilized surface.

The model described above was validated by performing SPR experiments using mutated/altered residues of IQ motif peptide (sequence provided in material & methods section). Initially we mutated first two residues of peptide (IQ to AA) but we could not find any significant change in Kd value. Further we mutated Arg731, Arg733 and Arg735 to Asp. The dissociation constant of mutated peptide with EhCaBP5 was calculated to be 4.1 mM as against 0.64 nM was observed with native peptide (Fig. 11). The observed dissociation constant shows that mutant IQ motif peptide binds to EhCaBP5 with less affinity and hence these positively charged residues Arg731, Arg733 and Arg735 are possible key amino acids that are involved in interaction and affinity with EhCaBP5, as indicated by the model.

## Discussion

One of the major interpretations of our structural studies is that the three-dimensional conformation of EhCaBP5 is more similar to that of myosin's ELC than to that of CaM, This was an unexpected finding as EhCaBP5 displays relatively high sequence similarity with CaM. This conformational similarity with ELCs is clearly seen on inspection as described in results and from the structure based alignment using the Dali server [25]. The most striking aspect of this similarity between EhCaBP5 and ELCs, but not with CaM, is that the EF-hand motifs of EhCaBP5 and ELCs are closed even in Ca^2+^ bound form.

The EhCaBP5 EF-hand motif remains in a closed conformation after Ca^2+^ binding even though the Ca^2+^ coordinating residues are canonical. The closed EF-hand motif conformation of EhCaBP5 can bind to the heavy chain and can stabilize the closed state of the whole N-terminal lobe through cooperative interactions with or without calcium. In CaM, the decrease in energy resulting from the binding of Ca^2+^ compensates for the increase in energy accompanying the conformational change that opens up the hydrophobic pocket. However, there are more hydrophobic residues on the helices of EhCaBP5 than on the helices of CaM and there are two calcium binding loops in CaM compared to the one site in EhCaBP5; these features cause an increase in the energy that would be needed to open the hydrophobic cleft of EhCaBP5. This is apparently not surmounted by binding of Ca^2+^ with the pocket remaining closed and the helices remaining stationary, the glutamate at the 12^th^ position of the EF-hand motif is positioned too far to coordinate the Ca^2+^. As a result, the Ca^2+^ bound EF-hand motif of EhCaBP5 is trapped in a so called intermediate state [20]. Our modelling of EhCaBP5 with myosin 1B IQ motif peptide suggests that EhCaBP5 adopts an extended conformation when it binds to myosin. Calmodulin like light chain of Mlc1p bound to IQ4 peptide of Myo2p also adopts an extended conformation, and it was expected that the extended conformation could mediate the formation of ternary complexes during protein localization and/or partner recruitment [26]. Therefore we expect that the extended conformation of EhCaBP5 with bound IQ motif may also allow for interactions with other molecular partners during various cellular processes.

The observation that the structure of EhCaBP5 resembles that of ELCs, expands the opportunity for studying this myosin heavy chain binding class of proteins. The importance of ELCs in regulating the function of myosins is well known. However, most of these studies have been carried out in just a few systems, notably mammalian, drosophila and *C. elegans*. Recently, one of myosin II light chain (CaBP20 or EAL50546) was identified [27] but there has been little information about ELCs and their regulatory role in myosin function in *E. histolytica*, especially on cellular myosin involved in phagocytic cup formation, and our functional studies of EhCaBP5 begin to address this issue.

A number of evidences suggest that the binding partner of EhCaBP5 is myosin 1B but not myosin II. Assays using purified molecules as well as cell extract based assays and cellular colocalization have been used to demonstrate this interaction. Over-expression and suppression of EhCaBP5 also influences the rate of phagocytic cup formation. Since EhCaBP3 was also shown to bind myosin 1B, it is important to compare differential role of these two myosin 1B binding proteins. Our imaging experiments clearly showed that these two myosin 1B binding proteins have different specific functions, though overall both participate in phagocytosis. While EhCaBP3 stays on the phagosomes even after separation from membrane, EhCaBP5 is found till phagosomes are getting closed, but absent in phagosomes after separation. EhCaBP5 is not involved in recruitment of myosin 1B while EhCaBP3 is [13] and EhCaBP3 does not bind to IQ motif (Figure S1). Moreover, EhCaBP3 binds F-actin and myosin 1B in the presence of Ca^2+^ [13], unlike EhCaBP5 that does not bind F-actin and does not require Ca^2+^ for interacting with myosin 1B. Furthermore, EhCaBP3 is also present inside the nucleus [13], a feature not displayed by EhCaBP5. These observations indicate both these calcium binding proteins are functionally different and that myosin 1B may be using different ELCs (or binding proteins) for carrying out different functions. Taken together, our structural studies show that EhCaBP5 resembles ELCs, and the functional studies indicate that it is likely to be the ELC of myosin 1B. *E. histolytica* has only two myosin in spite of high motility and tremendous high rate of phagocytosis. Other organisms, such as human has about 40 different myosin heavy chain genes, *Dictyostelium discoideum*, a closely related free living protist, encodes thirteen [28].

Therefore it is intriguing to understand how *E. histolytica* carries out all functions using only two myosins. We suggest that myosin 1B uses these two proteins as light chains to carry out different functions. If this is true then this helps partly to explain myosin paradox in *E. histolytica*.

The mechanism by which it dissociates from myosin before phagosomes are closed is not clear. Particularly it is difficult to explain at present given the slow dissociation rate of bound IQ motif peptide, and the role of Ca^2+^ in this process. It is possible that other yet unknown regulatory proteins may be involved in this process. In this report we have attempted to delineate the function of the calcium binding protein EhCaBP5 using both structural and cellular approaches and showed that it is a myosin 1B binding protein and participates in phagocytosis. EhCaBP5 is one of a number of growing calcium binding proteins (EhCaBP1, EhCaBP3, EhC2PK) that have been recently identified to be involved in amoebic phagocytosis suggesting that Ca^2+^ has an important signalling role in phagocytosis.

## Materials and Methods

### Preparation of selenomethionine labelled protein

Since the structure of EhCaBP5 (accession number EAL46660) could not be solved by molecular replacement [15], selenium-labelled protein was prepared to obtain phases. For preparation of selenium-labelled protein, *E. coli* BL21 (DE3) cells containing EhCaBP5 plasmid were grown overnight in LB media. Cells were harvested by centrifugation at 4000 rpm for 10 minute. Harvested cells were washed with selenomethionine medium (Molecular Dimensions) twice, to take out residual LB medium, and then was suspended in the same media for inoculation for the further culture as described before [29]. For protein expression and purification we have followed same protocol as describe earlier for native protein [15].

### Crystallization

Crystals of EhCaBP5 selenomethionine labelled protein were grown in similar conditions as were crystals of native EhCaBP5 [15].

### Data collection, processing and structure determination

SeMet- *Eh*CaBP5 crystals were soaked in cryoprotectant solution consisting of 2.8 M sodium acetate, 0.1 M Bis-Tris pH 5.5, and 20% glycerol. A single crystal was picked up in a cryo-loop and flash frozen in liquid nitrogen. A single wavelength anomalous dispersion (SAD) diffraction dataset was collected to 1.9 Å resolution at the Se edge (λ = 0.9788 Å) on a MARCCD 165 detector at the DBT-BM14 beamline of the European Synchrotron Radiation Facility (ESRF, France). The peak dataset was then indexed, integrated, and scaled using the HKL2000 [30]. Data collection statistics are shown in Table 1. The crystal belonged to space group C2 with unit cell parameters *a* = 70.55 Å, *b* = 44.45 Å, *c* = 47.73 Å, *α* = 90°, *β* = 108.9°, *γ* = 90°. Assuming one molecule of *Eh*CaBP5 per asymmetric unit, the crystal volume per unit of protein mass 2.32 Å^3^/Da [31], which corresponds to a solvent content of 47.3%. The sequence of *Eh*CaBP5 consists of four SeMet residues and the positions of the Se atoms were determined using SHELXD program (correlation coefficient, CC all/weak: 34.7/25.4; Patterson figure of merit, PATFOM 12.33) [32]. The initial phases were computed and partial model was built with SHELXE program as part of the HKL2MAP package [33], [34]. This partial model was used as a starting point for iterative automated model building and rebuilding along with sequence docking using Auto Build program in Phenix software [35], the remaining parts of the structure including side chains were modeled manually. The model was refined using the program REFMAC5 [36] and iterative manual rebuilding of the model was performed in COOT [37]. One Ca^2+^ atom was identified and included in the refinement. The translation-liberation-screw (TLS) displacement parameters were determined and TLS restrained refinement was performed [38]. For the final model, the *R*work is 18.7% and *R*free is 22.1%. The structure has good electron density (Figure S2) and stereochemistry as indicated by program PROCHECK [39] with 96.1% of residues lying in the most favoured regions of the Ramachandran plot. The final refinement statistics are shown in Table 1. The refined model of *Eh*CaBP5 and structure factors was deposited in the Protein Data Bank under the accession code 4OCI.

**Table 1:** Crystallographic data-collection statistics.

X-ray source	ESRF BM14 Beamline
Wavelength (Å)	0.9788
Space Group	C2
Cell parameters (Å, °)	a = 70.5; b = 44.4, c = 47.7
	α = 90, β = 108.9, γ = 90
Resolution range (Å)	20–1.9
B-factor Wilson plot (Å^2^)	25.6
Mosaicity range (°)	0.61–0.87
Total Reflections	63546
Unique Reflections	21006
Completeness (%)^a^^,^ b	98.7 (92.3)
Redundancy	3.0 (2.8)
Mean I/σ (I)	23.9 (2.13)
Rmerge(%)^b^^,^ c	7.5 (88.8)
ShelxD^d^: Data used (Å)	2.4
PAT figure of merit (FOM)	12.33
**Refinement statistics**	
Resolution range (Å)	20–1.9
Reflections used for refinement (all)	9684
Reflections used for *R*free	1212
*R*cryst(%)^e^	22.1
*R*free (%)	18.7
r.m.s.d. bond lengths (Å)	0.010
r.m.s.d.bond angles (°)	1.371

a Data completeness treats Bijvoët mates independently.

b Statistics for the highest resolution bin (1.9–1.93 Å) are given in parentheses.

c *R*_merge_ = ∑*_hkl_*∑*_i_*|*I(hkl)_i_*|−<*I(hkl)*>|/∑*_hkl_*∑*_i_*<*I(hkl)_i_*>.

d Substructure determination parameters are from ShelxD.

e *R*_cryst_ = ∑*_hkl_*||*F*_o_(*hkl*)|−*k*|*F*_c_(*hkl*)||/∑*_hkl_*|*F*_o_(*hkl*)|, where *F*_o_ and *F*_c_ are observed and calculated structure factors.

### Surface plasmon resonance (SPR)

The *E. histolytica* genome codes for two myosin, myosin I (accession number EAL48894) and myosin II (accession number EAL51645). Myosin I has one IQ motif and myosin II has two IQ motifs. To check the interaction between EhCaBP5 and myosin IQ motifs, we commercially synthesized (obtained) peptides of IQ motif of myosin 1B (unconventional myosin) (IQKAWKGYRNRKR) and second IQ motif of heavy chain myosin (myosin II) (LQACARAFAARKHFS), which is expected to bind to ELC. For the binding study we used Biacore T200 apparatus (Biacore, GE Healthcare) at National Institute of Plant and Genome Research New Delhi, India. A total of 2000 resonance units (RU) of peptides were immobilized on a research grade S series CM4 sensor chip in 10 mM sodium acetate, pH 5.0 according to the manufacturer's amine coupling kit. After peptide immobilization, the surface was blocked with 1 M ethanolamine at pH 8.5, followed by regeneration using 50 mM NaOH. The interaction experiments were performed using buffer containing 10 mM HEPES pH 7.4, 150 mM NaCl and 0.2 mM Calcium chloride. We also performed interaction experiment in absence of Ca^2+^ and supplementing 5 mM of EGTA in the above buffer. Binding experiments were carried with different concentrations (125,250, 500, 750, 1000, and 2000 nM) of EhCaBP5 in running buffer and injected at the rate 20 µL/min. For control we took bovine serum albumin (BSA) and EhCaBP3 at concentration 750 nM. The association kinetics for EhCaBP5 was monitored for 300 seconds and dissociation was monitored for the next 300 seconds.

To validate EhCaBP5-IQ motif complex model, we obtained commercially synthesized two mutated Myosin 1B IQ motif peptide IQ-M1 A*A*KAWKGYRNRKR (where IQ is mutated to AA) and IQ-M2 IQKAWKGYD*ND*KD* (Where R is mutated to D). The EhCaBP5 was immobilized on sensor chip up to 500 resonance units and native myosin 1B IQ motif peptide and mutated IQ motif peptide were passed as analyte at concentration of 25, 50, 75, 100 and 125 mM. The data were recorded at 25°C and data analysis was performed using Biacore T2000 SPR Kinetics evaluation software.

### Growth conditions, transfection and selection

*E. histolytica* stain HM1: IMSS and the transformants were maintained and grown in TYI-S-33 medium as described before [40]. Hygromycin (Sigma) were added at 10 mg ml^−1^ for maintaining transgenic cell lines as indicated. Transfection was performed by electroporation. Mid-log phase cells were harvested and washed first by PBS and then cytomix buffer (10 mM K2HPO4/KH2PO4 (pH 7.6), 120 mM KCl, 0.15 mM CaCl2, 25 mM HEPES (pH 7.4), 2 mM EGTA, 5 mM MgCl2). The washed cells were then re-suspended in 0.8 ml of cytomix buffer containing 4 mM adenosine triphosphate, 10 mM glutathione and 200 µg of plasmid DNA. The suspension was then subjected to two consecutive pulses of 3,000 V cm ^−1^(1.2 kV) at 25 µF (Bio-Rad, electroporator). The transfectants were initially allowed to grow without any selection for 48 h. Selection was carried out by adding hygromycin B (10 µg ml^−1^).

### Cloning of EhCaBP3S and EhCaBP3AS

pEhHYG-tetR-O-CAT shuttle vector was used for cloning of sense and anti-sense constructs. The CAT gene of pEhHYG-tetR-O-CAT [41] was excised using KpnI and BamHI and EhCaBP5 gene was inserted in its place in either the sense or the antisense orientation. The sequences of oligonucleotides used for making the above stated constructs are provided below,

CaBP5_sense_FP-5′CGGGGTACCATGCAAAAACACAATGAAGAC-3′

CaBP5_sense_RP-5′GCGGGATCCTTACTTGAAAACAGTCATTAATTG-3′

CaBP5_anti sense_FP-5′CGCGGATCCATGCAAAAACACAATGAAGAC-3′

CaBP5_ anti sense _RP-5′CCGGGTACCTTACTTGAAAACAGTCATTAATTG-3′

Standard molecular techniques were used for making all these constructs. These clones were transfected as indicated above.

### Immunofluorescence labelling

Amoebic cells were labelled as described previously [5]. Cells grown at 37°C for 48 h were first washed with PBS and then with incomplete TYI-S-33 medium. The cells were then resuspended in the same medium and were allowed to grow on coverslips at 37°C for 10 min followed by fixation with 3.7% formaldehyde for 30 min, washed with warm 1× PBS and permeabilized with 0.1% Triton X-100 for 5 min. Additional treatment using chilled methanol (−20°C) for 3 min was carried out for staining myosin 1B. Permeabilized cells were then washed with PBS and quenched with 50 mM NH_4_Cl for 30 min at 37°C, followed by blocking with 1% BSA-PBS for 1 h. The cells were then stained with primary antibody for 1 h followed by Alexa Fluor 488 conjugated or TRITC conjugated anti-mouse secondary antibodies.

F-actin was labelled with phalloidin using a similar protocol as above except the methanol step was omitted. Antibody dilutions used were: EhCaBP5 at 1∶200, EhCaBP1 at 1∶200, phalloidin (Sigma; 1 mg/ml) at 1∶250, myosin 1B at 1∶150, anti-rabbit or mice Alexa 488 (Molecular Probes, Catalogue No. A-11008 or A-11001) at 1∶200, anti-rabbit or mice Alexa 555 (Molecular Probes, Cat. No. A-21428 or A-21422) at 1∶300. The preparations were further washed with PBS and mounted on a glass slide using DABCO [1, 4-diazbicyclo (2, 2, 2) octane (Sigma) 10 mg/ml in 80% glycerol]. The edges of the coverslips were sealed with nail-paint to avoid drying. Confocal images were visualized by using an Olympus Fluoview FV1000 laser scanning microscope.

### RBC uptake assay

To quantify the red blood cells (RBC) ingested by amoebae, the colorimetric method of estimation was followed with little modifications [42]. Briefly, 1×10^7^ RBCs were washed with PBS followed by TYI-S-33 and then incubated with 1×10^5^ amoebae for different time points at 37°C in 0.5 ml culture medium. The amoebae and erythrocytes were pelleted and non-engulfed RBCs were lysed with cold distilled water and

Centrifuged at 1000 *g* for 2 minutes. This step was repeated twice, followed by resuspension in 1 ml formic acid to burst amoebae containing engulfed RBCs. The optical density of the samples was determined by a spectrophotometry at 400 nm using formic acid as the blank.

### Immunoprecipitation

Immunoprecipitation was carried out as described previously [13]. Briefly, CNBr-activated Sepharose-4B beads (Pharmacia) were conjugated with anti-EhCaBP5 antibody. Crude immunoglobulins were collected from the immunized serum using 40% ammonium sulphate and subsequently dialysed in coupling buffer (bicarbonate buffer). Usually, 10 mg immunoglobulin protein was added per gram of CNBr-activated Sepharose-4B beads. The resin was mixed gently for 18 h at 4°C. The conjugated Sepharose beads were incubated with *E. histolytica* lysate for 6 h at 4°C. The beads were then washed thrice with wash buffer (10 mM Tris-Cl (pH 7.5), 150 mM NaCl, 1 mM imidazole, 1 mM magnesium acetate, 2 mM β-ME and protease inhibitor cocktail). Ca^2+^ and EGTA were maintained throughout the process as required. After incubation the beads were washed sequentially with 60 mM Tris-Cl (pH 6.8), 100 mM NaCl and with 60 mM Tris-Cl (pH 6.8). The pellet was suspended in 2× SDS polyacrylamide gel electrophoresis (PAGE) buffer and boiled for 5 min followed by centrifugation for 5 min. The proteins were then analysed by western blotting.

### Modelling and docking of EhCaBP5 with IQ motif

Analysis of the crystal structure showed that the conformation of the Ca^2+^ bound EF-hand motif of EhCaBP5 resembles that of myosin ELC, the coarse grained model of the EhCaBP5-peptide complex was obtained using the crystal structure of squid myosin, which contains its ELC and associated IQ motif-containing heavy chain (PDB ID-3I5G) [18]. The peptide bound conformation of EhCaBP5 was obtained by employing the flexible superimposition protocol of RAPIDO structural alignment software [43]. The course grained complex model of CaBP5-IQ motif used as the starting structure was then used for the molecular docking simulations with the IQ motif peptide using Rosetta FlexPepDock web server [44]. The Rosetta FlexPepDock protocol optimizes the protein-peptide complex using Monte-Carlo algorithm along with energy minimization [45]. In this study we used 200 models for refinement and chose the best model based on their Rosetta generic full-atom energy score (Figure S3). The images were prepared using Pymol [46] visualisation software.

## Supporting Information

Figure S1 **EhCaBP3 was run on myosin 1B IQ motif immobilized surface at concentration of 750 nM.** The curve indicates that EhCaBP3 does not interact to myosin 1B IQ motif as the curve is closer to baseline.
(TIF)

Figure S2 **Electron density map of representative are of EhCaBPs at 1.5 σ cut-off.**
(TIF)

Figure S3 **Modelling of EhCaBP5 and IQ motif using FlexPepDock.** A) Shows the top 10 peptides superimposed at the binding site. B) A plot of the 200 models created by FlexPepDock, showing Rosetta score (y-axis) *vs.* RMSD from the reference structure (x-axis); rmsBB - RMSD is calculated only for peptide backbone heavy atoms.
(TIF)
