# Correction: Crystal Structure of Calcium Binding Protein-5 from Entamoeba histolytica and Its Involvement in Initiation of Phagocytosis of Human Erythrocytes

**DOI:** 10.1371/journal.ppat.1004716

**Published:** 2015-03-27

**Authors:** The PLOS Pathogens Staff

Fig. 11 is incorrect. The Native peptide Kd value should be 0.64nM. The authors have provided a corrected version here.

**Fig 11:**
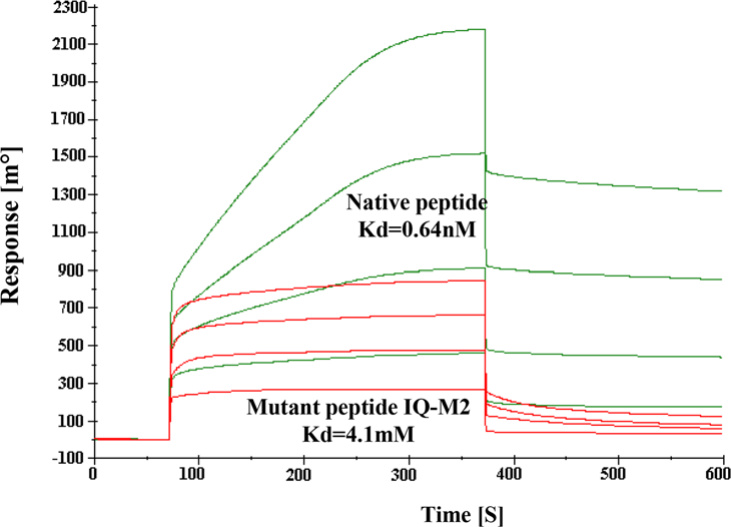
Model Validation.

SPR Sensogram represents the binding of Native IQ motif peptide (green) and Mutant IQ-M2 peptide (red) to EhCaBP5 immobilized surface.
