# Supplementary figures and images for: Crystal Structure of Calcium Binding Protein-5 from Entamoeba histolytica and Its Involvement in Initiation of Phagocytosis of Human Erythrocytes
Source: PLoS Pathog. 2014 Dec 11;10(12):e1004532. doi: 10.1371/journal.ppat.1004532 (PMC4263763; doi:10.1371/journal.ppat.1004532)

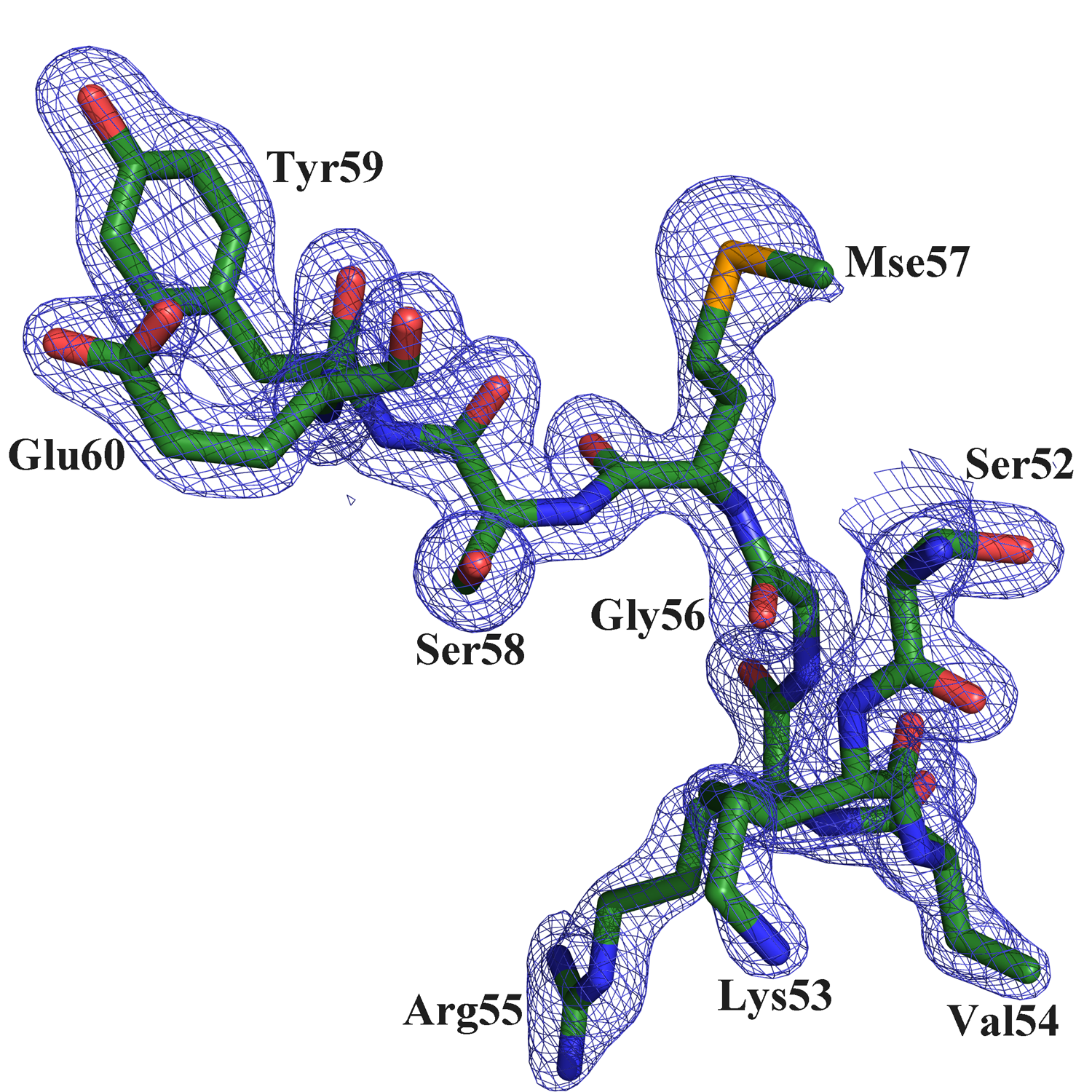

Supplement: Figure S1 — EhCaBP3 was run on myosin 1B IQ motif immobilized surface at concentration of 750 nM. The curve indicates that EhCaBP3 does not interact to myosin 1B IQ motif as the curve is closer to baseline. (TIF) [file ppat.1004532.s001.tif]

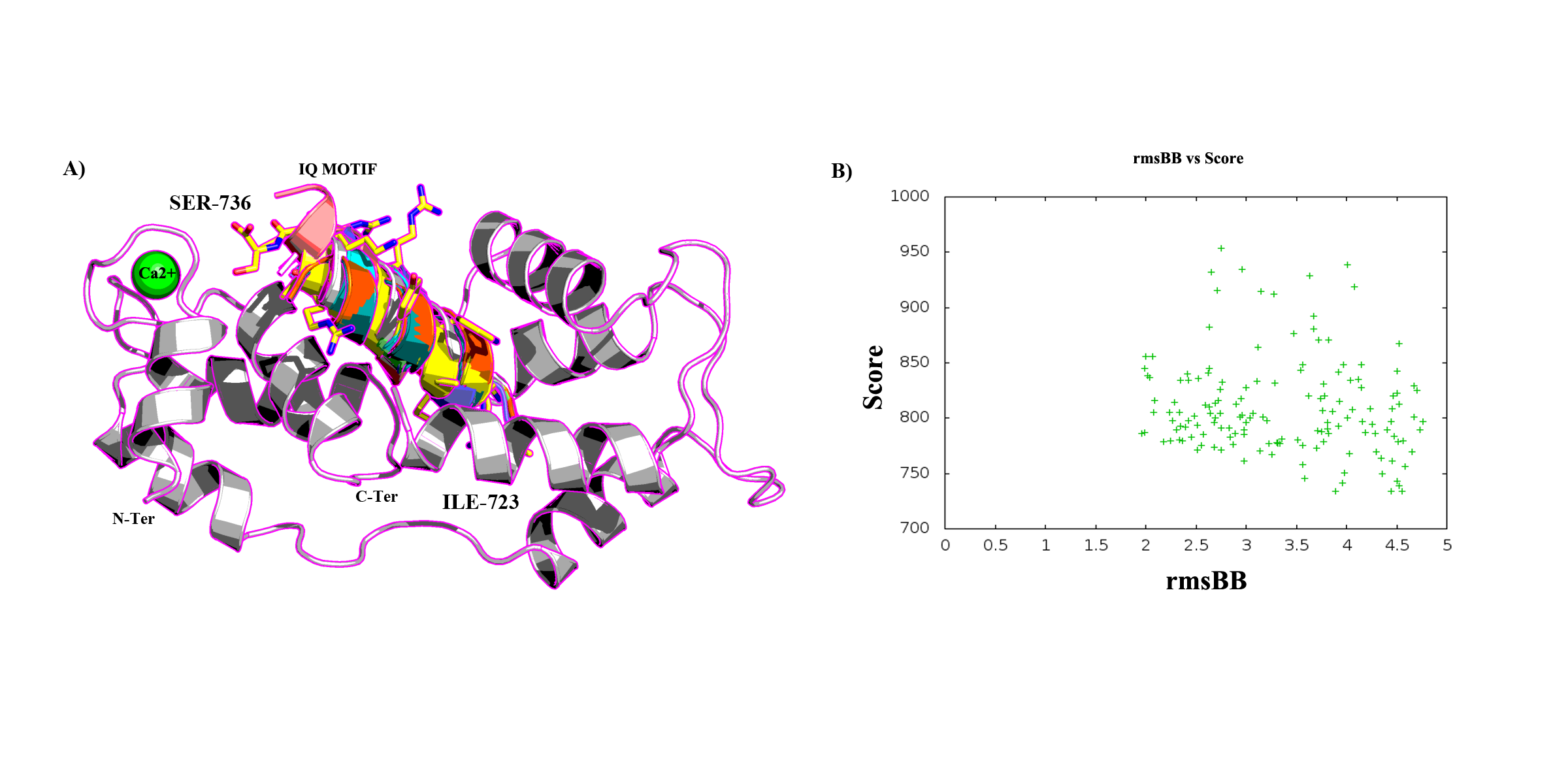

Supplement: Figure S2 — Electron density map of representative are of EhCaBPs at 1.5 σ cut-off. (TIF) [file ppat.1004532.s002.tif]

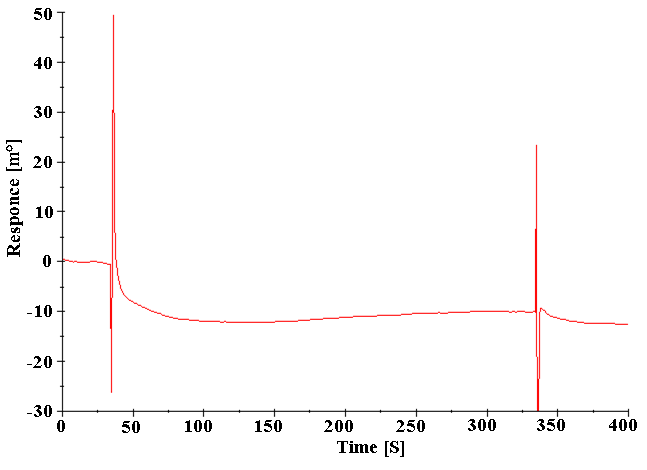

Supplement: Figure S3 — Modelling of EhCaBP5 and IQ motif using FlexPepDock. A) Shows the top 10 peptides superimposed at the binding site. B) A plot of the 200 models created by FlexPepDock, showing Rosetta score (y-axis) vs. RMSD from the reference structure (x-axis); rmsBB - RMSD is calculated only for peptide backbone heavy atoms. (TIF) [file ppat.1004532.s003.tif]
